# Beach Tourism in Times of COVID-19 Pandemic: Critical Issues, Knowledge Gaps and Research Opportunities

**DOI:** 10.3390/ijerph17197288

**Published:** 2020-10-06

**Authors:** Seweryn Zielinski, Camilo M. Botero

**Affiliations:** 1 Department of Hospitality and Tourism Management, Sejong University, 209 Neungdong-ro, Gwangjin-gu, Seoul 05006, Korea; 2 Grupo de Investigación en Sistemas Costeros, Playas Corporacion, Calle 19 8-44, Santa Marta 050022, Colombia; playascol@gmail.com

**Keywords:** SARS-CoV-2, coronavirus, beach management, tourist beach, crisis

## Abstract

The strict quarantine measures employed as a response to the COVID-19 pandemic have led the global tourism industry to a complete halt, disrupting the livelihoods of millions. The economic importance of beach tourism for many destinations has led many governments to reopen tourist beaches, as soon as the number of infection cases decreased. The objective of this paper is to provide a scientific basis for understanding the key issues for beach tourism management in these circumstances. These issues include risk perception, environmental considerations directly related to beaches and COVID-19, and management strategies designed to limit the risk of contagion on the beach. The contribution of this paper lies in its interdisciplinary approach to delivering the findings from the latest studies, highly relevant for beach tourism, in psychology, health science, and environmental science (often in preprint and in press format). Particular attention was given to identifying the knowledge gaps evident in the areas of COVID-19 risk perception, with the drivers explaining the risk-taking behavior and the protective strategies employed by beachgoers. Gaps were also found in areas such as the presence of SARS-CoV-2 in bathing waters and the sand, the potential of contaminated sand being a viable route of transmission, and the impact of the use of chemical disinfectants on the marine environment and on bathers. The paper identifies research prospects in these areas, additionally pointing out other questions such as new carrying capacity methods, the opportunity given by COVID-19 in estimation of the impacts of visitation and beach-litter.

## 1. Introduction

The global spread of the novel coronavirus SARS-CoV-2 causing severe acute respiratory syndrome (COVID-19) has resulted in social, medical and economic shocks that will be felt for years to come. The impact of the outbreak of the COVID-19 has been felt in many spheres, from international disputes among different countries, which further increased tense trading relationships, to economic stagnation or decline resulting in the collapse of many companies and unemployment [1]. Although, according to modelling research, the strict lockdown policies implemented between 2 and 29 March effectively decreased the spread of the virus averting an estimated 3.1 million deaths in 11 European countries [2], and prevented 530 million infections in in China, South Korea, Italy, Iran, France, and the US [3]; it also had devastating effect on economies.

Strict social distancing, temporary travel bans and, in some cases, more extreme quarantine measures such as complete lockdowns have caused economic losses estimated to reach hundreds of billions of dollars. The World Bank estimated under the normal scenario that globally GDP is expected to decline by 2.1% with an estimated decline of 2.5% in developing countries and 1.9% in high-income countries [4]. They also estimated the decline in global GDP would reach 3.9% under amplified global pandemic scenarios. More pessimistic estimates state that the world economy will shrink by 6% in 2020 [5]. The prognosis for the tourism sector alone is that there will be a decrease in output of 50–70% [2] and up to 80% by some estimates [6]. Because of the economic importance of tourism, the impact of COVID-19 on travel-related industries has been studied in many countries and destinations around the world; e.g., [7,8,9,10,11,12,13].

It is believed that the recovery from the crisis will not only have the aforementioned direct economic implications, but it will lead to a competition between countries for tourists caused by oversupply and not matching demand [14]. The countries that are recovering from the lockdown begin to offer incentives to attract tourists; e.g., [15]. However, a long-term recovery is expected to be slow and in the process of recovery many companies will not be able to survive [14]. The economic slow-down will also impede fast recovery of the demand for tourism as leisure spending of many families will be affected.

Although tourism is, in most countries, not the main economic driver, it is heavily relied on in some regions [16]. Tropical countries and those that depend on tourism throughout the year will be the most affected by the industry slowdown [17]. Empirical studies corroborate this notion. A research carried out on all American countries found that the highest risk category is principally comprised of small countries with expenditures from inbound tourism being much larger than their expenditures from outbound tourism (all of the countries of the Caribbean, with the exception of Haiti and Trinidad and Tobago) [9].

Other significant implication of COVID-19 can be found in the socio-cultural domain. The pandemic led to tensions as anti-Chinese rhetoric appeared based on China as the place of origin and spread of the virus [18]. For many tourism destinations around the world, the willingness for tourism to return is countered by the fear of potential virus transmission from tourists felt among local people. In this context, a study of three tourist cities in china (Hong Kong, Guangzhou, and Wuhan) shows that the residents are willing to pay for mitigation of the risk of COVID-19 contagion [19]. Another study looked at the opposite perspective to find out that 42.3% of tourist to Azores were willing to pay more for safer vacations [15].

The tourism industry has experienced various disruptive events in the past, none of which had a long-term negative impact on tourism [17]. Early evidence on the impact of COVID-19 suggests that its scale is much more extensive than with the earlier events. While many of the chain hotels located near beaches can afford a complete halt of activities while providing support (albeit limited) to their employees, the tourism sectors of many tropical developing countries present a greater challenge. A significant proportion of their workforce consists of informal workers [20] who are highly vulnerable to a sudden loss of income [21]. A reopening of the tourism industry is, therefore, unavoidable to protect the livelihoods and businesses.

For the many countries that offer “sun, sea and sand” (3S) tourism, beach tourism accounts for a significant amount of their total national revenue [22]. Given this, governments feel the pressure to reopen beaches at the first signs of a drop in COVID-19 cases and perhaps even sooner, although under strict monitoring and control measures. International travel is already resuming despite many countries still being amid the pandemic [23]. As restrictions on travel are lifted, the United Nations World Tourism Organization (UNWTO) emphasizes the need for responsibility and for prioritizing safety and security [24]. This is a crucial moment to strengthen governance through promoting cooperation among local governments, civil society and academia. There is a clear need for the involvement of experts in the development of strategies to respond to crisis events such as COVID-19 [23,24,25]. The objective of this paper is to provide a scientific basis for understanding the key issues relevant to beach tourism management in this context. These include tourists’ risk perception, environmental considerations directly related to COVID-19, and management strategies designed to limit the risk of contagion on the beach. The contribution of this paper lies in its interdisciplinary approach to deliver the latest findings (many accessed in their preprint and in-press versions), from psychology and health and environmental sciences that are highly relevant to beach tourism and the challenges presented by COVID-19. Particular attention was given to identifying knowledge gaps evident in the areas of COVID-19 risk perception, with the drivers explaining the risk-taking behavior and the protective strategies employed by beachgoers. Gaps were also found in areas such as presence of SARS-CoV-2 in bathing waters and the sand, the potential of contaminated sand being a viable route of transmission and the impact of the use of chemical disinfectants on the marine environment and on bathers. The paper identifies research prospects in these areas, additionally pointing out other questions, such as new carrying capacity methods, with the opportunity given by COVID-19 in estimation of the impacts of visitation and beach-litter.

## 2. Methods

An integrative literature review was chosen as an appropriate method that synthesizes secondary data about a wide range of topics directly relevant to tourist beaches. Two principle criteria were employed in the selection of papers to be included in the review. First, the papers had to present the latest empirical findings to provide the most up to date information. For this reason, many of the selected papers were in press format and were yet to be published. Second, only the papers in areas directly related to beach-user safety and the environmental hazard presented by SARS-CoV-2 were considered. Although a number of studies addressed some behavioral and environmental aspects of SARS and MERS in the past, the scale and characteristics of the SARS-CoV-2 transmission are different, which led the authors to believe that the social and environmental responses are different. Hence, this study intended to include principally the studies on COVID-19, integrating the findings from SARS and MERS studies as proxies only when COVID-19 studies were not available. Because researchers are often the ones advising authorities to open/close beaches in many parts of the world, this paper sought to identify relevant research to advance their own studies and knowledge on the most relevant topics.

## 3. Future Scenarios for Beach Tourism

With the continuing uncertainty about the impact of COVID-19, several possible scenarios are facing the tourism industry. These predictions range between a total collapse of the industry to an optimistic global switch to more sustainable consumption (see, for example, [26,27,28]). The most pessimistic scenario assumes that the extreme social perception of epidemiological risks seen in the first months of the pandemic will remain until the development of a reliable vaccine. In this scenario, the fear of infection creates a deep fear of social contact, which indisputably would have a devastating impact on beach tourism. Public fear would lead to a further loss of travel’s appeal and this would, in turn, force the authorities to increase restrictions on the use of beaches. Because of the high level of informality in the sector, this move would increase unemployment and cause consequent socio-economic conflicts because beach tourism would then be concentrated in private facilities considered safe [29].

At the other end of the spectrum, the optimistic scenario assumes a change in approach to tourism by the government and private sector [16]. The recovery from COVID-19 is most likely to overlap with the currently taking place, albeit slowly, global transformation of the current economic systems to those that have a net zero carbon footprint (carbon neutrality) [23]. As noted by Hall et al. [30], many industry experts thus optimistically assume that the pandemic presents a transformative moment or opportunity for a new opening leading to quicker adaptation of more sustainable environmental solutions [30]. The assumption that new beach tourism will emerge also assumes that a greater social appreciation of the natural and cultural value of beaches will emerge—an awareness reinforced by a collective reflection on the pandemic.

Many scientists and industry professionals see in the crisis a global opportunity to rethink old ways and reset the industry [27,28]. However, the early evidence shows that this is unlikely to be the case and that a return to business as usual [26] or possibly even increased growth to overcompensate for losses, is the most likely scenario [17,28]. Under this scenario, experts and scholars alike predict a domestic tourism recovery in the short term and a recovery of international tourism in the long term [28]. The shift to a carbon-neutral economy and the implementation of more sustainable practices is also unlikely because of the priority that will be given by governments and companies to the recovery from economic loss [13].

Several scholars and industry specialists have also predicted that demand for international beach tourism will be significantly reduced, but that this drop will be partially offset by domestic tourism [29] which is usually the first to recover after crisis [30]. Experts suggest that less accessible and isolated destinations will be perceived as having a lower risk of contagion and consequently will be preferred [25,31]. People are also more likely to reduce personal mobility, choosing their local environments that can meet their various aspirations [16,32]. They are likely to travel independently or in small groups to recover from self-isolation [25] and to avoid crowded places [33]. Beach users are likely to arrive earlier in the morning or later in the evening to avoid congestion and human contact [29].

It is still too early to confirm or reject the above predictions. Early evidence suggests that the pessimistic scenario is, at least partially, unlikely. The result of an online survey of 4000 respondents from 140 countries carried out by Bloom Consulting [31] states that 35 and 45%, respectively, declared that they would not travel unless the virus is either controlled or fully eradicated. The remaining 65% were willing to travel. The optimistic scenario seems equally unlikely. A transformation in tourism is possible only through changes in both the demand and supply side of the market [26]. Some beach destinations might, in the long term, make a switch to more sustainable consumption and some consumers might reassess their priorities. At present, however, news articles indicate a complete lack of change in the approach of tourists and the industry in some of the countries most affected by the COVID-19 crisis, such as the United States of America, Spain, Portugal, Canada, Brazil, and the United Kingdom (UK) [34,35,36,37,38]. This is not only the case with respect to domestic tourism. In Spain, local governments in various famous beach destinations had to close their beaches because of the arrival of tourists from the UK [39]. Because of the long period of isolation and warm summer weather in the northern hemisphere, thousands of people have ignored social distancing and government recommendations and have flocked to local beaches in great numbers [38].

## 4. Understanding Beachgoer Risk Perception

The decisions made by tourists about their chosen activities and travel destinations are influenced by their perceptions of safety and security [40]. Risk perception is a subjective psychological construct that is influenced by cognitive factors (knowledge and understanding of risks), emotional and experiential factors (personal experience), and social, cultural, and individual factors such as gender, education, and ideology [41,42,43,44]. In general terms, studies show that the more risk people perceive the less likely they are to visit public places such as beaches and the more likely they are to cooperate with government recommendations and to adopt health-protective behaviors [42,45,46,47,48].

As of the time of writing, various studies had been published on the perception of the risk of contagion by COVID-19. Dryhurst et al. [42] provided an international analysis of COVID-19 risk perception amongst 6991 individuals surveyed across ten different countries. The determinants of an individual’s risk perception were their experience with the virus, social amplification (hearing about the virus from friends and family), pro-sociality, individualistic worldviews, self and collective efficacy, trust, and personal knowledge. Other researchers identified the voluntariness of taking the risk to be another factor that negatively affects the perception of risk [41].

Wise et al. [48] surveyed 1591 individuals in the United States of America (USA). The results show that in the early stage of the pandemic subjects perceived their risk of being infected as relatively high and as even higher five days later when the number of infections in the country increased. Interestingly, people rated the average person in the USA as having the highest risk of infection, but themselves as having the lowest risk (optimism bias). Similar results were obtained by Gerhold [49] in the UK and Kuper-Smith [50] and the UK, USA and Germany. In both cases, those who reported lower-perceived risk also reported low engagement in protective behaviors and information seeking. De Bruin and Bennett [45] explored the perception of the likelihood of infection and subsequently fatality and found that the median perception of the risk was higher in the case of infection than in case of infection fatality. This was consistent with the results of a similar study by Gerhold [49]. The study also confirmed the finding observed by Wise et al. [48] that the perception of risk increased with time as a result of increases in the number of infections and/or information campaigns.

The above-mentioned studies suggest that besides the cultural factors, beachgoers are likely to underestimate their risk. This may be due to the voluntary nature of taking the risk, optimism bias, poor social amplification, lack of reliable information or having no direct experience with the virus. Although the perception of risk of infection is relatively high among the general population, the perceived risk of fatality is much lower, especially among younger people who are most likely to visit a beach. This is consistent with studies on adolescent risk-taking (e.g., [51,52]). Because closed indoor spaces are perceived to present the highest risk for virus transmission, while outdoor places are perceived to be safer [49], beaches are likely to be considered safe.

Studies also show that when the number of positive cases and fatalities decreases, the perception of risk may also decrease. The perception of risk is also likely to decrease in the absence of significant changes in the number of infected cases. As explained by habituated action theory, this takes place where there has been prolonged exposure to the risk without a negative outcome [53,54]. On the contrary, when the situation gets worse, the perception of risk may increase [48,49]. This would explain the mass beach visitation in the UK and other locations. When the rate of coronavirus deaths and new infections fell considerably and the lockdown restrictions were eased, the risk perception among beachgoers dropped dramatically “as if the last three months of fear and caution over the coronavirus vanished overnight” [38]. The most concerning for beach management in times of pandemic is the consistency of studies confirming a positive relationship between risk perception and the likelihood of engaging in preventive measures. If people who go to beaches do not perceive the risk, they are also likely to ignore preventive measures. This then further entrenches a general community perception that the activity is low-risk—a result explained by social action theory [52]. The need for management strategies that are effective and controllable is thus indisputable.

## 5. Environmental Considerations

This section of the paper is dedicated to environmental considerations brought to light by COVID-19. There have been several unexpected environmental impacts that might have dire consequences on beachgoer safety and entire beach tourism destinations. The sudden desertion of tourist beaches has had a short-term positive environmental impact on coastal environments that have had time to recover from human pressures [55,56,57]. The resulting visual and acoustic qualities of undisturbed coasts have been noted in the media (see, for example, [58,59]) and by early empirical research papers that confirmed these observations [57]. It is currently unknown whether these changes are substantial and long-term.

Besides the obvious temporal consequences, the pandemic also brought about various threats. Because of the high level of risk perceived by some individuals, it is safe to assume that the number of visitors to rural and natural beaches—which are usually less congested—will rise. Even a relatively small increase in visitors to beaches that are highly susceptible to anthropic pressures will have a greater environmental impact than a similar increase on urban beaches [60]. The increased visitation rate may cause not only an overload of scarce public facilities on these less visited beaches but may also encourage the construction of public infrastructure and facilities to cope with the demand and to ensure visitors’ safety (this might include parking, mechanical cleaning, and lifeguarding). These changes would have a significant environmental impact [61,62].

The potential impact wastewaters being infected by SARS-CoV-2 is highly relevant in the developing country context [63]. Despite the hostile conditions of the marine environment, viruses can maintain their infectious capacity in the water for prolonged periods, reaching the aquatic environment via sewage discharges [64,65]. They can also remain on solid particles suspended in the water column or on sand particles on the beach [66]. According to a recent review study by Kitajima et al. [67], an increasing number of studies have demonstrated the presence of SARS-CoV-2 in stool from patients confirmed as having COVID-19. This discovery indicates yet another route of contagion—fecal transmission. Preliminary results confirmed the presence of the virus in raw sewage [68,69,70,71]. The lack of treatment of domestic sewage and the discharge of rainwater directly or indirectly through rivers to the sea is a serious threat to the people utilizing that water in many coastal locations [72,73]. These people may get infected through either ingestion, inhalation, or dermal contact. Most bathing water monitoring programs include an evaluation of the concentration levels of microorganisms from fecal pollution (enterococcus and *E. coli*) [66]. On many tourist beaches, these levels surpass the maximum permitted thresholds after rainy days when rainwaters mixed with untreated or partially treated sewage enter the sea [74,75]. Despite the bacteriological monitoring carried out by local authorities on many tourist beaches, there are currently no protocols for the detection of SARS-CoV-2 in the water or sand on a beach.

The second environmental consideration that should be closely analyzed by local authorities and beach managers is the impact of the chemicals that are used in huge quantities to disinfect public spaces [76]. The conventionally used quaternary ammonium surfactants and their derivatives are capable of inactivating the SARS-CoV-2 virus [77]. However, these substances also affect the marine organisms when transported to the ocean via sewage and rainwater drainage systems [78]. They can cause an ecotoxicological impact that could reduce the populations of some animal groups (e.g., algae, fish, mollusks, barnacles, rotifers, starfish, shrimp and others) [79,80]. Some of these organisms are responsible for removing significant amounts of organic matter, as well as chemical and biological pollutants [77]. This would harm the environmental quality of beaches, which is a critical point in attracting visitors [60].

Finally, there is an issue of increased usage of protective gear such as gloves and masks that are often inappropriately disposed of [56,81]. These items when discarded can be transported to the sea by wind or rainwater either directly or indirectly through rivers, which are considered one of the main sources of beach-litter [82,83]. In coastal areas without good rainwater treatment, these potentially contaminated items are likely to end up in the sea and/or be washed out on beaches presenting a hazard to marine organisms such as crustaceans, mollusks, birds, turtles and fish that ingest microplastics from degraded synthetic material [84]. The presence of these items has already been reported in different locations on the planet [85,86,87]. However, it should be also noted that the absence of beachgoers, one of the main sources of litter on beaches [88,89], has a contrary effect. An evaluation carried out on Brazilian beaches showed lower than usual concentrations of plastics and other waste typically found in the dry strip of sand where most bathers are usually set up [90]. Similar findings were presented by Ormaza-González and Castro-Rodas [57] in the case of Ecuadorian beaches.

## 6. Strategies to Deal with COVID-19 on Tourist Beaches

Integrated beach management must address not only the immediate challenges of ensuring the safety of beachgoers and the appropriate environmental conditions to reopen beaches, but should also include long-term strategies that respond to the uncertainties posed by a prolonged pandemic (Table 1). It should be recognized that the most appropriate strategies will differ depending on the beach type (natural, urbanized or resort) and local conditions [91]. Because the paper focuses on issues related to COVID-19, many general beach management strategies are not discussed.

Currently, several interesting, albeit often unfeasible, ideas to ensure beach-user safety are circulating online. Beach booths made of plexiglass isolating beach-user groups from each other were reported to have made an appearance in Santorini, Greece [92] and to have been considered on various Italian beaches [93]. Although acrylic sheets withstand exposure to high levels of UV light, saltwater spray, and significant temperature changes without shattering, fogging or losing transparency, the idea is unrealistic. The booths are costly, reach unbearably high temperatures inside even with an open roof, and their daily cleaning requires considerable effort and chemicals. On the other hand, some more realistic voluntary measures have been proposed to deal with the requirement of social distancing on a beach. The most common is social distancing tapes or circle templates that are used to mark off an area around beach users [94] which are being sold on some beaches in Spain [95].

### 6.1. General Beach Visitor Management Strategies

Individuals who have the COVID-19 virus may be presymptomatic or asymptomatic. To avoid transmission in these cases, the bulk of management strategies center on ensuring social distancing. At most beaches in the world that still allow beach visitation, toilets have been closed to minimize the risk of skin contact with potentially infected surfaces. Because beach users tend to concentrate in areas with a wide range of facilities such as drink or snack stalls, kiosks, showers, etc., their closure or separation is recommended. In urban and resort beaches, social distancing can be achieved through a greater separation of deckchairs, parasols, beach huts or tents for rent. The beaches that use wooden catwalks for visitors’ movement on the beach should provide two lanes separated by at least a 2-m gap to limit close contact with those passing in opposite directions. Finally, when full protective measures cannot be implemented on the entire beach, a zoning system may be considered, separate areas for different user groups with stricter measures only in areas designated for high-risk groups such as the elderly.

Commonly employed in the hospitality sector are strategies related to service delivery such as the separation of tables, provision of mostly outdoor dining spaces, and requiring online reservations. Chinese tourists who experienced the 2003 SARS epidemic also employed voluntary measures such as ordering delivery or takeout food [25], although this can also increase waste generation. Finally, local governments can opt for strategies centered on spreading tourists across different locations by promoting beach alternatives or developed beaches.

### 6.2. General Beach Resort Management Strategies

In many places around the world, the beach product is inseparably related with beach resort operations. According to the preliminary findings of a longitudinal study by Gursoy and Chi [96], hotels are likely to see a slower post-pandemic recovery due to a reluctant attitude of potential customers (over 50% of respondents) to dine and stay in hotels. However, as the industry is recovering, it is important to highlight a number of strategies that are applicable to these indoor spaces. Because hotels and tourism accommodations are equally susceptible to contagion any indoor space visited by large numbers of people and a high degree of interaction, besides the general prevention measures that are usually compulsory for all hotels such as frequent hand washing, physical distancing, wearing a mask and frequent disinfection procedures [97,98], the response from hotel managers should include a set of more specific strategies.

Based on the experiences from the SARS pandemic, scholars point out the value of having a disaster management plan with standard operating procedures (SOPs) that can greatly enhance the effectiveness of the response [99,100,101]. Existing SOPs that do not include epidemiological risk should be adjusted and implemented in accordance with the recommendations of local and national public health authorities with the aim to prevent cases, effectively manage cases, and mitigate impact among clients and staff, including cleaning and disinfection of rooms occupied by ill persons [98,102]. It is advisable to keep a logbook of actions and measures carried out that can be evaluated and used to further improve the SOP [100].

Because the reception desk staff belong to the highest risk of infection group, if possible, staff members should not be older or with underlying health conditions. Reception desk employees should be equipped with disinfectant/wipes for surface cleaning, disposable gloves and apron, full-length long-sleeved gown and biohazard disposable waste bag [102]. The general recommendations of the WHO [102] for hotels state that they pay a special attention to disinfecting surfaces that are frequently touched such as handrails, elevator buttons, handles, doorknobs, computer keyboards, key cards, writing utensils, toilets, handwashing basins, baths, etc. [98]. Guests should avoid handling food at the buffets and special care should be given to the disinfection of the commonly used surfaces such as drink dispensers after each use. The concentration of water disinfectants in swimming pools should be kept at the upper recommended limits. The WHO also recommends to have a maximum of four persons for 10 square meters and separation of sitting guests of at least 1 m. Consideration should be given to closing the recreational areas for children, as well as other public areas and facilities such as gyms [100].

Resorts should provide guidelines to the staff on how they should communicate the action plan to guests. Informative posters and leaflets can amplify the key messages among guests and staff. Communication strategies not only increase the awareness about the risk, but also decrease their fear and increase confidence among visitors and staff members [103,104]. Moreover, the resort staff should undergo the training required for understanding and adopting the measures that could protect their health and that of others [98,100,102]. It is especially important because many standard procedures that has been followed for years are likely to change (cleaning, washing, operating cafeteria, communication with guests, etc.).

Technology is seen as an alternative solution to deal with the actual and perceived risk of the virus contagion in the hotel industry [96,97,100,103]. The internet-based communication technologies are currently used in hotels around the world at the management level for work-related communication and customer interaction with hotel staff and facilities (self-check-in, voice control of room service, etc.) [100]. However, the implementation of more advanced technologies such as infrared scanners, app-based keyless doors, touchless elevators, digital menus, automatic water/soap/drink dispensers, kiosk check-in machines, robot cleaning systems, electrostatic sprayers, ultraviolet-light technology, and advanced heating, ventilation, and air conditioning (HVAC) systems [96,97,100,103], dependent on the “good will” of hotel owners and the availability of appropriate resources [97]. According to a recent study, both restaurant (64.71%) and hotel (70.42%) customers indeed believe that these technologies will be necessary to minimize human-to-human contact, and many of them (30–40%) are even willing to pay more for increased safety precautions [105]. Other empirical studies confirm this notion stating that the technology-mediated solutions lead to lower levels of perceived health risk among hotel visitors [103].

### 6.3. Environmental Management Strategies

The absence of data about the possibility of SARS-CoV-2 contagion through seawater, and a lack of protocols for the detection of viruses in the water and sand on a beach means that a precautionary approach is recommended. The established relationship between fecal bacteria and viruses, including SARS-CoV-2, requires that bathing should be banned on beaches exposed to significant discharges of untreated sewage and rainwater runoff. On urban beaches that record high levels of fecal contamination after rainfalls, a no-bathing period of at least 72 h is recommended after rain [64]. In the absence of water-quality monitoring, access to the sea should be closed until more information about the virus and fecal contamination is available.

In the early stage of the pandemic, when a timely response was vital, public authorities scrambled to secure any type of disinfectant substances. There is evidence, albeit anecdotal, of the use of highly toxic substances such as sodium hypochlorite (bleach) to disinfect public beaches [106]. The only beach cleaning methods that are recommended are the aeration of sand to allow the UV light to act as a natural disinfectant [107] or the adding of ozone to mechanical beach cleaners to protect the sand from bacteria and viruses [108]. In terms of the disinfection of other public spaces, public authorities should carefully review the usage of substances based on quaternary ammonium surfactants that can enter the sea through rainwater runoff.

### 6.4. Carrying Capacity

The concept of beach carrying capacity (BCC) has become particularly relevant. Commonly criticized as imprecise to calculate and hard to control, it is still one the most important tools for visitor management in beach environments [109]. BCC allows authorities to determine the maximum beach load based on user densities or availability of parking spaces. The BCC is especially important as it allows a calculation of the space, measured in square meters, required by visitors for their psychological comfort (psychological carrying capacity).

The BCC concept has been criticized as difficult to implement due to limits on the ability of authorities to control the number of visitors on multiple-access public beaches. The suppression of parking spaces has been the most common strategy to limit capacity [109]. However, due to the restrictions posed by the COVID-19 pandemic, stricter control measures are not only advisable but also widely acceptable. For control over the number of visitors a “one entrance/exit” strategy is recommended, along with measures to create separate lanes of people and limit close contact between people passing in opposite directions. When establishing the maximum user density using BCC factors to consider are the usable beach area, visitors’ spatial patterns (visitors tend to use the first 30m from the sea) [110], and social groupings (social “bubbles” requires less space overall). Once established, the BCC can be managed through a reservation system, as has been proposed in countries such as France and Spain, to control the number of visitors to their mostly urban and resort beaches [111,112]. The alternative to reservations is a tracking system such as that implemented in Belgium that indicates the density of visitors on specific beaches and reroutes them to less populated areas [111].

The social distancing of visitors on the beach can be controlled by implementing roped-off grids varying in the area and based on party size [112] or through the voluntary measures such as distancing tapes described earlier. Enforced separation of user groups may not be required as long as the total number of visitors on a beach is controlled and there is enough space; even before COVID-19, beachgoers usually voluntarily practiced social distancing through crowd-avoidance self-regulation [110]. Social carrying capacity studies on beaches established that, depending on the beach type, beachgoers require an area of between 8 m^2^ and 22 m^2^ per user for a pleasant experience [110,113,114]. The lower threshold, applied mostly to urban beaches, is likely to increase given that psychological comfort depends on a beach user’s level of perceived risk in different stages of the pandemic. When calculating the BCC, it is thus advisable to consider both the physical distancing and psychological comfort.

### 6.5. Information and Education

Protection motivation theory states that engagement in protective measures is significantly influenced by high levels of perceived risk [45,46]. Because perceived risk is influenced by the knowledge and understanding of risks by the people who perceive it and on social amplification [42], obtaining information from a variety of sources, such as the government and media, is of crucial importance [47]. Public authorities have to work closely with media outlets to share knowledge and build confidence to reduce fear [41]. Transparency about scientific uncertainty does not undermine public trust [115], hence “clear communication of risk could aid the development of accurate risk perception, in turn facilitating engagement in protective behaviours” [48] (p. 10). The provision on the beach of information about the virus itself and of the measures taken by beach management to ensure safety are two strategies designed to regaining visitor confidence. Signage with information on the risk of contagion and recommendations for appropriate behavior, including the beach’s carrying capacity and allowed beach-user density (if controlled), should be displayed. Risk perception studies show that there is a subset of individuals who do not seek out information and can only be targeted by direct outreach methods such as emergency alerts on cellphones [48].

### 6.6. Beach Management Organization

The objective of a beach management organization (BMO) is to regulate the interrelations between different elements in a coastal system intended to achieve a balance between economic activities and the environmental health of the beach [116]. A BMO assumes the central decision-making role, coordinating the often conflicting interests of stakeholders and mediating conflicts when necessary. In a way, BMOs are similar to destination management organizations (DMOs), but because they operate on a much smaller scale they are more focused on beach management and conflict resolution and much less on marketing and destination-level tourism planning. The integration of institutional and private stakeholders into a BMO allows for greater coordination of decisions about crisis response strategies that are both suitable and as fair as possible to a variety of beach stakeholders. These strategies include the implementation of a rotation system for the operation of beach microenterprises (e.g., food stalls, peddlers, and equipment rentals) ensuring an equal opportunity to earn at least a partial income. The benefit of the active involvement by a variety of stakeholders and local communities is that they are all then more likely to assume personal and collective responsibility for applying response measures [117].

BMOs, especially on certified beaches, can also exercise their institutional power by pressuring government agencies to provide funds and to take specific actions [118] to ensure the best possible response to the pandemic—including such measures as providing protective gear, hiring more staff, and involving volunteers. Other relevant advantages of BMOs is their ability to generate cooperative relationships between small and medium beach enterprises and beach peddlers to take advantages of economies of scale in purchasing goods and protective gear and thus maximize their potential income [29].

## 7. Knowledge Gaps and Research Needs

In times of crisis, resources are allocated to new priorities and research teams around the world scramble to develop science-based solutions to ensure that the most effective measures are taken. The coronavirus pandemic introduced many new and complex problems for which the scientific community has no answers. Scholars have already called for interdisciplinary approaches to deliver scientifically grounded feasible solutions [25] that would increase the safety of citizens and at the same time provide a viable way to engage in familiar tourism and leisure activities. In the context of beach tourism, there are many gaps in knowledge that need to be addressed to ensure a safe beach experience. This section focuses on these gaps in the hopes of sparking interest within the scientific community to research in a safe and precautious manner using such methods as online surveys and scientific webinars.

COVID-19 risk-perception studies have confirmed that individuals who perceive less risk are less likely to cooperate with government recommendations and to adopt protective measures [25,42,45,46,47]. Given the evidence that beaches still attract thousands of visitors even in countries that are experiencing increasing COVID-19 cases, there is a need to investigate risk perceptions, with the drivers explaining the risk-taking behavior and the protective strategies employed by beachgoers. Researchers may want to understand why a subset of individuals do not engage in seeking information and protective measures to develop direct outreach methods on beaches.

In terms of environmental considerations, beach safety must include investigating the presence of SARS-CoV-2 in bathing waters and the sand, especially on those beaches that receive untreated or partially treated domestic sewage. On the other hand, because viruses are known to have the ability to survive for extended periods on sandy beaches under conditions of low light and high humidity, there is a need to estimate the lifespan of SARS-CoV-2 in beach-type conditions and to consider the efficiency of sand aeration and UV solar radiation in deactivating the virus. Currently, there is still no information on the ability of the virus to remain viable in seawater, although early evidence from Spain indicates this presents a low risk [119]. Likewise, no studies have been published about contaminated sand being a potential route of transmission. Research gaps are also evident in the field of beach ecotoxicology [76]. Further studies are required on the impact of the use of chemical disinfectants and possibly safer biological alternatives on the marine environment and on bathers who are exposed.

Given the recent media coverage, it seems that many coastal ecosystems have recovered quickly, within a few months of the halt of tourism activities. Whether this recovery has been substantial remains unknown. Before the pandemic, the possibility of investigating the resilience of many intensively used beaches was very limited. There is now also an opportunity to estimate the impact of visitation by comparing the environmental conditions of partially recovered beaches (the baseline) with their post-pandemic condition when visitation resumes. A very similar opportunity is presented in the field of beach-litter assessment. The proportion of marine litter that can be attributed specifically to beach users has been very difficult to estimate as there are many possible litter sources [120]. There is a considerable amount of data regarding litter on extensively used tourist beaches collected during the summer high season that cannot easily be compared with data collected in the low season when environmental conditions are very different [121]. The existing summer data can, however, be compared with new data collected on beaches that, because of COVID-19, are relatively empty during the same period. Additionally, the pandemic’s impact on the quantity of marine litter washing up on beaches can be estimated.

The beach carrying capacity tool also requires further research attention as it remains the primary means of calculating the maximum allowed number of beach users at any given time. The conditions presented by COVID-19 require a re-evaluation of the tool. Health and safety recommendations and environmental factors need to be taken into consideration. Now, more than ever, the estimation of a beach’s social carrying capacity (SCC) is of the utmost importance as it takes into account the beach users’ perceptions of risk as relevant to their perceived safety and hence the overall quality of the experience. There is also an evident need for research into the addition of a new component to the carrying capacity calculation that is centered on the epidemiological risk posed by COVID-19. The epidemiological carrying capacity (ECC) should consider the space required by each beach-user group to limit contagion but should also consider the issue of beach access. Provision should be made for well-spaced one-way entrance, exit and walking paths to limit the chance of people coming into close contact with one another as they move about. Additionally, given the propagation characteristics of SARS-CoV-2, the calculation of the ECC at a beach should consider social groupings or bubbles rather than only individual users. The visitor part size could be calculated by analyzing data from the reservation systems that are becoming a popular means for controlling visitor numbers. The grid system being implemented on many beaches should take into account both the ECC and the SCC, as these components are likely to determine the final carrying capacity—except perhaps in the case of highly natural or protected beaches. Further research is required on visitor-flow patterns and SARS-CoV-2 transmission characteristics to guide new methods for calculating beach carrying capacity.

## 8. Limitations of the Research

The research has two major limitations that stem from the fact that the SARS-CoV-2 pandemic is still a recent phenomenon and the body of relevant research, although growing fast, is still limited. Therefore, the findings from a limited number of studies may not be conclusive and contradictory results from future studies can be expected. Although the majority of the topics addressed in this paper and not new and were addressed previously in relation to the SARS and MERS pandemics, the scale and characteristics of SARS-CoV-2 transmission are different, which is likely to produce different social and environmental responses. For this reason, the studies carried out on SARS and MERS in the past that were used in this research as proxies in the absence of SARS-CoV-2 research may not be completely accurate.

## 9. Conclusions

The SARS-CoV-2 epidemic has already had unprecedented social consequences causing economic losses estimated to reach hundreds of billions of dollars. Of the possible future scenarios, the evidence suggests that a sustainable reopening of the tourism industry is unlikely. Rather, and more pessimistically, it seems inevitable that the priority given to economic recovery will lead to the more pessimistic business as usual scenario. Many governments have already decided to reopen tourist beaches, even without a drop in COVID-19 levels. Considering the economic importance of 3S tourism for many destinations around the globe, governments, civil society and academia alike must act to develop and implement viable solutions to face the crisis. In the interests of more quickly answering the key pandemic-related questions facing beach tourism destinations, this paper has focused on the latest findings from various disciplines highly relevant to beach tourism, pointing out the knowledge gaps and research opportunities.

The studies of COVID-19 risk perception demonstrated that there is a clear relationship between an individual’s likelihood of cooperating with government recommendations and their adopting health-protective behaviors. Outside of cultural factors, beachgoers are likely to underestimate their real personal risk because of the voluntary nature of taking the risk, optimism bias, poor social amplification, a lack of reliable information, and no direct experience with the virus. These findings are especially relevant in the context of public spaces where the risk of contagion is high.

The pandemic has also highlighted various, in some cases unexpected, environmental threats, impacts and concerns. These include the impact of the increased number of visitors to rural and natural beaches, the contamination of bathing waters by the virus, the lifespan of the virus on the sand, the impact of chemicals used for disinfection, and the pollution caused by discarded protective gear.

Various strategies can be used to deal with these threats including imposing a bathing ban on beaches exposed to significant discharges of untreated sewage and rainwater runoff, reviewing the use of certain disinfectants, and using aeration and sand ozonation techniques. There is a range of strategies for beach-user safety centered on ensuring the physical distancing of visitors and management of their risk perception. These include closure or separation of certain facilities, providing new service delivery options, using online reservation systems, promoting alternatives to the beach, implementing a strict carrying capacity, providing information to manage risk perception, and establishing a beach management organization. Though some of these strategies have proven to be effective, many have never been implemented and tested in COVID-19 conditions.

The future lines of research should be focused on investigating risk perceptions specifically in the context of COVID-19 as it has proven to have different transmission rates and hence perceive risk from the previous viruses (SARS, MERS). The drivers explaining the risk-taking behavior, protective strategies employed by beachgoers, and measures to develop direct outreach methods on beaches also require investigation. In relation to the environmental considerations, the presence of SARS-CoV-2 in bathing waters and the sand and the potential of contaminated sand being a viable route of transmission, as well the impact of the use of chemical SARS-CoV-2 disinfectants on the marine environment and on exposed bathers, require further investigation. Finally, studies that adapt the concept of carrying capacity to meet the safety requirements are warranted. With COVID-19 becoming a new normal, researchers have to act quickly to confront its challenges.

## Figures and Tables

**Table 1:** Management strategies to deal with COVID-19 on beaches.

General visitor management strategies	Closure of public toilets Closure or separation of amenities such as drink or snack stalls, kiosks, showers Separation of deckchairs, parasols, beach huts or tents for rent Provision of two unidirectional wooden catwalks to separate beach users moving in opposite directions Implementation of a zoning system for high-risk groups of users Promotion of food delivery service Provision of sparsely located dining spaces Spreading visitors among different beaches or alternative locations
	Promotion of the usage of social distancing tapes or circle templates
Environmental management strategies	Closure of bathing for at least 72 h on urban beaches that record high levels of fecal contamination after rainfalls Ban on highly toxic substances for beach disinfection Aeration of sand to allow the UV light to act as a natural disinfectant Addition of ozone to mechanical beach cleaners to protect the sand from bacteria and viruses Careful usage of substances based on quaternary ammonium surfactants
Carrying capacity	Control over the number of visitors (first come-first served/online reservation) Separation of beach entrance and exit Usage of social bubble concept and spatial distribution patterns when calculating carrying capacity Implementation of a grid system to manage distance between visit groups Implementation of social carrying capacity based on perception of risk
Information and education	Provision of information about COVID-19 risk and protective measures on the beach Implementation of direct outreach methods such as emergency alerts on cellphones
Beach management organization	Creation of a beach management organization that integrates relevant stakeholders Implementation of a rotation system for small enterprises on the beach
